# Health education pathway for individuals with temporary enterostomies using patient journey mapping

**DOI:** 10.1515/med-2025-1317

**Published:** 2025-12-17

**Authors:** Sha-Sha Qin, Yan-Hua Liu, Su-Qing Chen

**Affiliations:** Department of Gastrointestinal Surgery, Shanxi Bethune Hospital, Shanxi Academy of Medical Sciences, Tongji Shanxi Hospital, Third Hospital of Shanxi Medical University, Taiyuan, Shanxi Province, China

**Keywords:** health education, patient journey mapping, satisfaction, self-care ability, temporary enterostomy

## Abstract

**Objectives:**

This study aimed to evaluate the effectiveness of a structured health education pathway for patients with temporary enterostomies, guided by patient journey mapping.

**Methods:**

Sixty-six patients undergoing temporary enterostomy in 2024 were enrolled. Patients were assigned to either conventional health education (control group) or a structured pathway based on patient journey mapping (experimental group). Outcomes assessed included patient satisfaction, health knowledge, self-care ability, and postoperative complications.

**Results:**

Compared with the control group, the experimental group demonstrated significantly postoperative satisfaction, health knowledge, and home self-care ability (p<0.05), and a lower incidence of complications (p<0.05).

**Conclusions:**

A personalized health education pathway guided by patient journey mapping effectively enhances patient satisfaction, knowledge, and self-care while reducing postoperative complications, supporting recovery in individuals with temporary enterostomies.

## Introduction

In China, more than one million individuals are living with an ostomy, and the number of new cases increases by approximately 100,000 annually. Temporary ileostomy accounts for approximately three-quarters of all ostomy cases [1]. Temporary enterostomy (TE) is a crucial intervention in the management of colorectal cancer, serving as a temporary excretory diversion created in the proximal bowel to divert intestinal contents away from the anastomotic site [2]. However, stoma-related complications occur in approximately 50 % of individuals with TE, and 3–25 % of these patients require permanent stoma placement due to severe complications that prevent enterostomy closure [3]. The disruption of normal bowel function presents significant physical, psychological, and social challenges [4]. Following surgery, individuals with a stoma typically undergo three phases of adjustment: acceptance, adaptation, and autonomy [5].

Studies indicate that health education plays a critical role in enhancing self-care ability and reducing the incidence of complications among individuals with TE. However, in clinical practice, there is a high demand for ostomy care knowledge, while traditional health education models often fail to meet patient needs due to a lack of systematic structure and continuity. In one department, the health knowledge awareness rate among individuals with TE was reported to be only 10 %. Addressing the comprehensive and continuous health education needs of individuals undergoing TE is essential for successful enterostomy closure. Patient journey mapping is a visual tool used to identify key stages, experiences, and patient needs throughout the disease trajectory [6]. Therefore, developing and implementing a structured health education pathway guided by patient journey mapping represents a critical strategy for improving postoperative satisfaction, increasing health knowledge awareness, reducing complication rates, and enhancing home-based self-care ability.

In recent years, patient journey mapping has been increasingly applied in the management of chronic diseases. This approach helps to optimize the understanding of patient needs, identify pain points and challenges in the care process, and thereby improve both the care experience and patient satisfaction. When applied to individuals with intestinal stomas, patient journey mapping can further identify timely opportunities for intervention and provide patients with diverse and targeted educational support.

## Materials and methods

### Research participants

This study employed a historical control design. The control group comprised 33 individuals who underwent temporary enterostomy at a tertiary hospital in Taiyuan City between January and June 2024. The experimental group included 33 individuals who underwent the same procedure at the same hospital between July and December 2024.

The inclusion criteria were as follows: (1) age between 18 and 70 years; (2) intact consciousness, effective communication ability, absence of cognitive dysfunction, and ability to perform activities of daily living independently; (3) signed informed consent for the use of clinical biological information; and (4) absence of severe comorbidities. The exclusion criteria were: (1) end-stage disease with a survival expectancy of ≤ six months and (2) presence of major organic lesions. No significant differences were observed between the two groups in terms of sex, age, marital status, education level, or employment status (p>0.05) (Table 1).

**Table 1:** Baseline characteristics of the two groups.

Characteristics		Control group, n=33	Experimental group, n=33	χ^2^	p-Value
Age	18–45 years	6 (18.18)	7 (21.21)	0.58	0.748
	> 45–60 years	13 (39.39)	15 (45.45)		
	> 60 years	14 (42.42)	11 (33.33)		
Sex	Male	20 (60.61)	18 (54.55)	0.248	0.618
	Female	13 (39.39)	15 (45.45)		
Marriage	Married	25 (75.76)	27 (81.82)	0.363	0.834
	Unmarried	4 (12.12)	3 (9.09)		
	Widowed/divorced	4 (12.12)	3 (9.09)		
Educational level	Primary school	6 (18.18)	7 (21.21)	1.074	0.783
	Middle school	10 (30.30)	12 (36.36)		
	High school	15 (45.45)	11 (33.33)		
	Bachelor’s degree and above	2 (6.06)	3 (9.09)		
Employment status	Employed	13 (39.39)	15 (45.45)	0.58	0.748
	Retired	14 (42.42)	11 (33.33)		
	Unemployed	6 (18.18)	7 (21.21)		

### Methods

#### Control group

Individuals in the control group received conventional health education provided by the department, which included face-to-face education, verbal explanations, graphical materials, and questionnaire assessments, without obtaining timely feedback on patients’ level of acceptance. Relevant patient information was collected.

#### Experimental group

In addition to the conventional health education provided to the control group, a structured health education pathway based on patient journey mapping was implemented for the experimental group. Personalized education was provided according to key challenges, intervention points, evolving patient needs throughout the disease trajectory, and the specific conditions and requirements of each individual. The aim of this approach was to identify service gaps, enhance awareness of ostomy care, and optimize the overall patient experience.

The following measures ensured that the education plan developed for patients was both feasible and effective:

(1)
**Targeted and Simplified Educational Content:** ① Addressing actual needs: Before developing the education plan, patients’ primary health concerns (e.g., common symptoms, medication precautions) were identified through questionnaires and interviews. Educational content was then designed around these core needs, avoiding irrelevant or overly complex information. ② Simplification of medical terminology: Professional medical terms were translated into plain, easy-to-understand language. Visual aids such as images, charts, and diagrams were used to further enhance comprehension.
(2)
**Diversified Educational Approaches:** ① Face-to-face communication: Healthcare staff conducted one-on-one or small-group sessions with patients. The pace and approach were adjusted in real time based on patient feedback, with immediate clarification of unclear points. ② Video-based education: Short, engaging science popularization videos (5–10 min) were produced or selected and shared via patient WeChat groups, allowing patients to access the materials at their convenience. ③ Printed materials: Well-structured brochures with clear layouts and visual illustrations were distributed. The content focused on key knowledge points and step-by-step procedures, enabling patients to review the material repeatedly at home.
(3)
**Evaluation and Adjustment of Educational Effectiveness:** ① Knowledge testing: Simple quizzes (e.g., multiple-choice or true/false questions) were administered before and after the educational intervention to assess changes in patients’ understanding of key medical knowledge. Results were analyzed to identify areas of insufficient comprehension, which were then re-explained. ② Behavioral observation: Patients’ health-related behaviors in daily life were monitored. For those who did not achieve the expected behavioral improvements, underlying reasons were explored, and the educational plan was adjusted accordingly.

The personalized education conducted in the experimental group was structured as follows: (1) Defining individualized education goals: Objectives were tailored according to each patient’s learning ability, personal interests, and specific nursing challenges to ensure targeted outcomes. (2) Planning educational content: The content was adjusted based on patients’ educational backgrounds. For those with lower education levels, simple and easy-to-understand instructions for basic procedures were provided; for patients with higher education levels, more in-depth explanations were added, such as intestinal anatomy, to enhance deeper understanding. (3) Selecting teaching methods: A variety of approaches were adopted, including one-on-one guidance and group-based collaborative learning, where patients were grouped to facilitate peer communication and mutual learning.

##### Establishment of the temporary ostomy journey mapping research team

A multidisciplinary research team was formed, comprising one attending colorectal surgeon with 10 years of clinical experience, two ostomy therapists with over 10 years of experience (holding at least a bachelor’s degree or a senior nursing title), and two specialist nurses with more than five years of clinical ostomy nursing experience and a nurse practitioner title or higher. All team members participated in training sessions focused on temporary ostomy journey mapping, covering topics such as surgical techniques for temporary stoma creation, standardized stoma care procedures, identification and management of common ostomy-related complications, key aspects of postoperative monitoring, selection of ostomy appliances, and daily postoperative care. The training was conducted through a combination of online and offline clinical practice sessions.

To ensure timely provision of health management services based on patient journey mapping, specific roles were assigned within the team. Clinicians were responsible for diagnosing and managing disease-related conditions and handling medical emergencies. Ostomy therapists oversaw the management of peristomal complications, follow-up of complex stomas, and documentation of patient needs and concerns at different stages of the journey. Specialist nurses were responsible for collecting general patient data, conducting follow-up assessments, and gathering questionnaire responses.

##### Health education pathway for individuals with temporary enterostomy based on patient journey mapping

(1)
**Admission stage:** Advancements in surgical techniques have led to an increased anus-preserving rate in individuals with low rectal cancer, resulting in a rise in the number of temporary stomas created postoperatively. Upon admission, an assessment of the individual’s health status was conducted, and interview data were analyzed. At this stage, individuals were primarily concerned with the surgical procedure and subsequent treatment plan. Many patients experienced anxiety during the waiting period, including fear related to the stoma, potential cancer metastasis, mortality, or concerns about body image. To address these concerns, nurses and physicians provided timely explanations regarding the surgical method and its necessity, as well as shared successful case examples to enhance confidence in disease management. The nurse in charge introduced pre-rehabilitation strategies, including preoperative exercise training, nutritional support, psychological interventions, and guidance in stopping smoking. Other aspects of care followed the standard procedures used for the control group.
(2)
**Preoperative stage:** In addition to general health education, personalized strategies were tailored to individual’s knowledge level, psychological state, and preferred learning methods. Through direct communication, healthcare providers identified specific health education needs, provided timely guidance, and helped alleviated anxiety. Family members were encouraged to participate in the patient journey mapping process to offer emotional support. Both patients and their families were informed in advance about postoperative recovery strategies and rehabilitation plans.
(3)
**Postoperative stage:** Building upon general health education, semi-structured interviews revealed that individuals often experienced stigma related to dependency and uncertainty about safe movement, fearing ostomy bag leakage and imposing additional burden on healthcare providers and family members. To address these concerns, healthcare professionals provided timely explanations of the surgical procedure and ostomy bag replacement techniques while guiding individuals on proper movement techniques. Early mobilization was encouraged to facilitate bowel function recovery. The initial change in bowel function was often distressing; therefore, individuals were instructed to use a small mirror to observe the stoma’s position, shape, and color, gradually fostering acceptance. Additionally, individuals were informed that the stoma closure would typically occur within 3–6 months and that the situation was temporary, which helped enhance motivation for postoperative recovery.
(4)
**Pre-discharge stage:** Self-care ability was assessed to ensure that individuals could perform ostomy care independently or with minimal assistance. Pre-discharge interviews indicated that many individuals expressed concerns about managing complications at home and the lack of access to professional ostomy care compared to the hospital setting. To address these concerns, detailed discharge instructions were provided, covering diet, daily activities, social interactions, and exercise rehabilitation. Individuals were informed about potential post-discharge complications, provided with the department’s contact information, and instructed to seek medical guidance if needed. Regular follow-up appointments were scheduled.
(5)
**Rehabilitation period and preparation for ostomy closure:** Regular follow-ups were conducted at one week, one month, and three months, supplemented by ongoing home-based guidance. Interviews during this phase revealed a strong desire for ostomy closure and high expectations for the procedure. Any peristomal skin complications that could affect closure were promptly addressed. Dietary guidance was provided to encourage a light, easily digestible diet while maintaining stable ostomy function and ensuring adequate nutritional support. Additionally, pelvic floor muscle exercises were recommended to improve anal sphincter contractility in preparation for normal bowel function following the second surgical procedure. The closure procedure and its preparatory steps were explained to individuals, enhancing their engagement in the recovery process.

#### Evaluation indicators

##### Nursing satisfaction

The Patient Satisfaction Questionnaire (PSQ) was administered to evaluate satisfaction levels. The questionnaire assessed six dimensions: the professional and technical competence of medical staff, the service attitude of medical staff, the method of health education delivery, the continuity of health education, and the attention given to individuals’ psychological needs. A Likert scale was utilized for scoring (1–5, where 1 represents “dissatisfied” and 5 represents “very satisfied”). The specific scoring criteria were as follows: 1 point indicated dissatisfied, 2–3 points indicated slightly satisfied, 3–4 points indicated moderately satisfied, and > 4 points indicated very satisfied.

##### Health knowledge awareness

Health knowledge awareness was assessed using a department-developed questionnaire covering five dimensions: stoma types and functions, identification and management of complications, preparation and procedures for ostomy material replacement, perioperative pre-rehabilitation exercise training, and dietary requirements. The questionnaire had a total score of 100 points, with≥90 points indicating mastery, ≥ 60 points indicating basic mastery, and < 60 points indicating a lack of mastery. It evaluated individuals’ understanding of the disease, essential pre- and postoperative knowledge, and enterostomy-related information. Higher scores reflected a greater awareness of relevant health knowledge.

##### Stoma self-care ability

The self-care ability of individuals before and after the nursing intervention was assessed using the Exercise of Self-Care Agency (ESCA) Scale. This scale consisted of 43 items divided into four dimensions: self-care skills, self-care responsibility, self-care perception, and health knowledge level. Each item was rated using a 5-point Likert scale, with 32 items scored positively and 11 items scored in reverse. The total score was 172 points, with higher scores indicating greater self-care ability. The scale demonstrated good reliability in our study sample. The internal consistency coefficients for the four dimensions were as follows: Self-Concept 0.81, Self-Care Skills 0.83, Self-Care Responsibility 0.84, and Health Knowledge Level 0.79. To further verify reliability, we also calculated the more robust McDonald’s ω coefficients: Self-Concept 0.82, Self-Care Skills 0.84, Self-Care Responsibility 0.83, and Health Knowledge Level 0.82. All coefficients exceeded 0.70, indicating that the scale has reliable internal consistency in our study population.

##### Incidence of ostomy complications

The incidence of stoma-related complications, including ischemia of the stoma mucosa, stoma stenosis, ostomy bag leakage, and peristomal moisture-associated dermatitis, was compared between the two groups from the time of surgery to ostomy closure.

#### Data collection methods

Specialist nurses were responsible for data collection, which included general patient information, the satisfaction questionnaire, the health knowledge questionnaire, the self-care ability scale, and the ostomy and peristomal skin complication questionnaire. Data were gathered through one-on-one interactions in person, via WeChat, telephone, and ostomy follow-up platforms at multiple time points: one day before surgery, three days after surgery, at discharge, and post-discharge (at one week, one month, and three months). The purpose, content, and time required for each questionnaire were explained in advance to ensure that individuals understood the objective and provided informed cooperation. If a participant did not complete the questionnaire on the scheduled day, a specialist nurse followed up by telephone the next day to determine the reason and document any loss to follow-up. Semi-structured interviews were also conducted with participants in the experimental group via face-to-face, WeChat, or telephone interactions. Data from each follow-up were compiled by specialist nurses and provided to ostomy therapists for statistical analysis. All participants were followed up as scheduled before undergoing ostomy closure surgery.

#### Statistical methods

SPSS software was used for statistical analysis. The t-test and chi-square test were employed, with statistical significance set at p<0.05. For complication rates, the Fisher exact probability method was applied.

Several measures were used to minimize temporal risk. We ensured that participant recruitment across different groups occurred within a relatively concentrated time frame to reduce the impact of long-term temporal variations. Additionally, we conducted a comprehensive baseline assessment and matched groups as closely as possible on key demographic and clinical variables to account for potential time-related differences in participant characteristics.

### Ethics approval and consent to participate

This study was conducted with approval from the Ethics Committee of Shanxi Bethune Hospital. This study was conducted in accordance with the declaration of Helsinki. Written informed consent was obtained from all participants.

### Consent for publication

Not applicable.

## Results

### Nursing satisfaction

Six aspects were evaluated in the survey: the professional and technical level of medical staff, the service attitude of medical staff, the method of providing health education, the continuity of health education, the effectiveness of health education, and the attention given to the psychological needs of individuals receiving care. The findings indicated that the proportion of individuals reporting moderate to high satisfaction in the experimental group was significantly higher than in the control group, with a statistically significant difference (p<0.05) (Table 2).

**Table 2:** Comparison of nursing satisfaction scores.

Satisfaction levels	Control group, n=33	Experimental group, n=33	Z	p-Value
Dissatisfied	16 (48.48)	9 (27.27)	−2.307	0.021
Slightly satisfied	8 (24.24)	5 (15.15)		
Moderately satisfied	6 (18.18)	12 (36.36)		
Very satisfied	3 (9.09)	7 (21.21)		

### Health knowledge awareness

Health knowledge awareness was evaluated across five dimensions: type and function of the stoma, identification and management of complications, preparation and procedures for ostomy material replacement, perioperative pre-rehabilitation exercise training, and dietary requirements. The results indicated that the health knowledge awareness score in the control group was 7.82 ± 2.64 before the intervention and 11.33 ± 2.29 after the intervention, whereas the experimental group had a score of 7.58 ± 2.41 before the intervention and 13.61 ± 2.69 after the intervention. The difference was statistically significant (p<0.001) (Table 3).

**Table 3:** Comparison of health knowledge awareness scores.

Items		Control group, n=33	Experimental group, n=33	Z	p-Value
Type and function of the stoma	Mastery	3 (9.09)	5 (15.15)	−2.289	0.022
	Basic mastery	8 (24.24)	16 (48.48)		
	Not mastery	22 (66.67)	12 (36.36)		
Identification and treatment of complications	Mastery	2 (6.06)	6 (18.18)	−2.095	0.036
	Basic mastery	9 (27.27)	13 (39.39)		
	Not mastery	22 (66.67)	14 (42.42)		
Preparation and process of replacing the ostomy materials	Mastery	1 (3.03)	3 (9.09)	−2.966	0.003
	Basic mastery	6 (18.18)	16 (48.48)		
	Not mastery	26 (78.79)	14 (42.42)		
Perioperative pre-rehabilitation exercise training	Mastery	1 (3.03)	6 (18.18)	−2.009	0.045
	Basic mastery	9 (27.27)	11 (33.33)		
	Not mastery	23 (69.70)	16 (48.48)		
Dietary requirements	Mastery	2 (6.06)	9 (27.27)	−2.41	0.016
	Basic mastery	12 (36.36)	13 (39.39)		
	Not mastery	19 (57.58)	11 (33.33)		

### Stoma self-care ability

The self-care ability of the two groups before and after the nursing intervention was evaluated using the ESCA Scale. The assessment included four dimensions: health knowledge level, self-care perception, self-care responsibility, and self-care skills. The results indicated that the experimental group achieved significantly higher scores across all four dimensions compared to the control group, with a statistically significant difference (p<0.05) (Table 4).

**Table 4:** Comparison of self-care ability scores.

Items		Control group, n=33	Experimental group, n=33	t_between-group_	p-Value_between-group_
Health knowledge level	Before intervention	17.21 ± 3.40	17.79 ± 3.62	−0.666	0.508
	After intervention	19.33 ± 2.47	21.61 ± 2.30	−3.865	<0.001
	t_in-group_	−0.754	−4.790		
	p_in-group_	0.003	<0.001		
Self-care concept	Before intervention	9.52 ± 2.08	9.48 ± 2.18	0.058	0.954
	After intervention	11.76 ± 2.85	13.52 ± 2.59	−2.623	0.011
	t_in-group_	−3.917	−6.294		
	p_in-group_	<0.001	<0.001		
Self-care responsibility	Before intervention	20.39 ± 3.44	20.70 ± 3.63	−0.348	0.729
	After intervention	23.24 ± 2.85	25.91 ± 2.99	−3.706	<0.001
	t_in-group_	−3.560	−7.024		
	p_in-group_	0.001	<0.001		
Self-care skills	Before intervention	38.70 ± 3.99	39.21 ± 3.81	−0.536	0.594
	After intervention	41.97 ± 2.82	43.76 ± 2.57	−2.688	0.009
	t_in-group_	−3.856	−5.350		
	p_in-group_	<0.001	<0.001		

### Complication rate

A comparison of peristomal complications between the two groups from surgery to ostomy closure indicated that the p-value for stoma stenosis and ostomy bag leakage was 1, indicating no significant difference between the two groups. This finding may be attributed to the type of surgical procedure performed and the extent of preoperative preparation. However, the total incidence of ostomy-related complications in the experimental group was significantly lower than in the control group, with a statistically significant difference (p<0.05) (Table 5).

**Table 5:** Comparison of ostomy-related complication rates.

Complications	Control group, n=33	Experimental group, n=33	χ^2^	p-Value
Ischemia of the stoma mucosa	5 (15.15)	2 (6.06)	0.639	0.424^a^
Stoma stenosis	4 (12.12)	3 (9.09)	0	1.000^a^
Ostomy bag leakage	4 (12.12)	3 (9.09)	0	1.000^a^
Peristomal moisture-associated dermatitis	8 (24.24)	3 (9.09)	2.727	0.099
Total ostomy complications	21 (63.64)	11 (33.33)	6.066	0.014

^a^Fisher’s exact probability method is used.

## Discussion

### Advantages of implementing patient journey mapping in the health education pathway for individuals with temporary enterostomy

The International Agency for Research on Cancer (IARC) reports approximately 1.9 million new cases of colorectal cancer annually, accounting for 10 % of all newly diagnosed cancers worldwide [7]. A temporary enterostomy is a critical intervention in the surgical treatment of intestinal diseases, particularly colorectal cancer. Although ostomy care education is primarily delivered in hospital settings, patients often lack direct professional support after discharge, and the knowledge and skills acquired during hospitalization require ongoing reinforcement. Insufficient proficiency in ostomy self-care may lead to a higher incidence of complications and may negatively affect the feasibility of timely ostomy closure. The implementation of a health education pathway guided by patient journey mapping allows healthcare professionals to assess the experiences of individuals with a temporary enterostomy from multiple perspectives. This approach facilitates the identification of key transitional stages, evolving needs, and challenges throughout the disease course, thereby providing a foundation for targeted interventions [8].

### Impact on the incidence of complications

Advancements in medical technology and an increased emphasis on quality of life have resulted in a growing preference for anus-preserving procedures among individuals undergoing rectal cancer surgery. Prophylactic temporary enterostomy reduces the incidence of postoperative anastomotic leaks in individuals with colorectal cancer. However, approximately 50 % of individuals with a temporary enterostomy experience stoma-related complications, which differ from those associated with permanent ostomies [9]. During hospitalization, ostomy-related knowledge is primarily acquired through direct education from healthcare professionals. Upon discharge, however, the lack of continuous professional guidance increases the risk of peristomal complications. Traditional health education approaches often fail to address post-discharge challenges effectively, highlighting the need for a more structured and comprehensive educational framework. The health education pathway guided by patient journey mapping enables the timely identification of challenges faced during home-based care, allowing for targeted interventions through communication platforms such as WeChat groups and video consultations [10].

Findings from the present study indicate that the implementation of this educational approach was associated with a reduction in the incidence of mucosal bleeding and peristomal moisture-associated dermatitis. However, no significant improvement was observed in the occurrence of stoma stenosis or ostomy bag leakage. This may be attributable to factors such as surgical technique and preoperative stoma site marking. These findings indicate the need for further investigation into optimizing preoperative planning and post-discharge support strategies.

### Effect on health knowledge awareness and self-care ability

Patient journey mapping, as described by Barton et al., is a tool that captures an individual’s interactions with healthcare professionals at different stages of a disease or therapeutic approach [11]. With the increasing emphasis on enhanced recovery after surgery (ERAS), hospital stays for individuals with an ostomy have shortened. Consequently, the opportunities for individuals and their caregivers to receive comprehensive training and guidance have become more limited. Insufficient exposure to ostomy care education during hospitalization often results in inadequate self-care proficiency upon discharge, contributing to gaps in health knowledge and self-management skills [12].

The findings of this study indicate that the implementation of a structured health education pathway under patient journey mapping significantly improved health knowledge awareness and self-care ability among individuals with a temporary enterostomy. The effectiveness of this approach may be attributed to its ability to systematically identify the evolving physiological and psychological needs of individuals throughout the disease course. By providing individualized and targeted health education interventions, this approach ensures that individuals receive the necessary support to develop confidence and competence in self-care.

### Effect on satisfaction at discharge

In the context of the high-quality development of public healthcare institutions, patient satisfaction has become a critical metric for evaluating the effectiveness of medical services. A patient-centered approach has increasingly been recognized as the cornerstone of healthcare delivery, emphasizing the importance of enhancing the overall patient experience [13]. Improving nursing services and optimizing patient experience will continue to be central to the advancement of hospital-based healthcare systems in China in the coming years. Patient journey mapping facilitates improves communication between individuals and healthcare professionals, promotes patient-centered care, and enables the systematic assessment of experiences within complex and dynamic healthcare settings [14].

The findings of this study indicate that the integration of patient journey mapping into health education pathways effectively captures and adapts to the evolving needs of individuals with a temporary enterostomy. By providing structured educational interventions, this approach enhances health knowledge awareness, improves self-care ability, reduces the incidence of complications, and ensures timely ostomy closure. Consequently, this comprehensive approach contributes to increased patient satisfaction and an improved overall healthcare experience.

This study has several limitations. First, data collection was limited to a single source, and both data acquisition and follow-up were retrospective in nature, which may have introduced bias. Second, the sample size was relatively small, the study duration was short, and the coverage was not comprehensive. In addition, this study relied on patient self-reported data. While validated instruments were employed, self-reports are susceptible to recall bias and response bias. Also, as this study was non-blinded, patients’ subjective perceptions may have influenced their responses, potentially introducing bias into the results. Therefore, further research is needed to explore patient-specific characteristics, nurses’ acceptance of care strategies, and the impact on the overall quality of patient care.

## Conclusions

In summary, the implementation of a health education pathway for individuals with temporary enterostomy, guided by patient journey mapping, demonstrates that this systematic and continuous educational approach can significantly enhance self-care ability and quality of life, reduce the incidence of complications, and improve nursing satisfaction. However, despite its advantages, this study has certain limitations. First, the sample size was relatively small, and stratified education based on varying educational levels was not incorporated. Second, the extended time span of the survey may have introduced recall bias or experience dilution, as participants were asked to reflect on past experiences. Additionally, the post-ostomy closure phase was not included in this study. These findings highlight the need for further research incorporating multifaceted and targeted investigations to develop more precise and comprehensive patient-centered strategies.

## References

[1] Lei WA, Gu YH, Yue Y, Xie CT, Chou RX (2022). Analysis on status quo of resilience in patients undergoing temporary ileostomy and its influencing factors. Shanghai Nurs..

[2] Li DY, Zhuang JY, Lin HY, Lin N, Zhu R, Wang Y (2024). Summary of the best evidence on health education for patients with temporary enterostomies. Chin Nurs J.

[3] Pérez Domínguez L, García Martínez MT, Cáceres Alvarado N, Toscano Novella A, Higuero Grosso AP, Casal Núñez JE (2014). Morbidity and mortality of temporary diverting ileostomies in rectal cancer surgery. Cir Esp.

[4] Becker MAJ, Pronk AJM, Gecse K, Hompes R, Bemelman WA, Buskens CJ (2025). Long-term outcomes of ‘temporary’ defunctioning in patients with severe perianal crohn’s disease. Colorectal Dis.

[5] Zhang Y, Xian H, Yang Y, Zhang X, Wang X (2019). Relationship between psychosocial adaptation and health-related quality of life of patients with stoma: a descriptive, cross-sectional study. J Clin Nurs.

[6] Dai MQ, Liao XQ (2024). Advances in patient journey mapping in the care of patients with chronic diseases. Nurs J.

[7] Norte A, Martínez C, Pasalodos A, Tort I, Sánchez A, Hernández P (2024). Impact of the laparoscopic approach, early closure and preoperative stimulation on outcomes of ileostomy closure after rectal resection. Cir Esp.

[8] Wen YJ, Cai TT, Niu N, Liu J, Li RY, Song ZH (2024). Study on the health management journey map of young and middle-aged colorectal cancer patients with colostomy. Chin Nurs Manag.

[9] Jabbal IS, Spaulding AC, Lemini R, Borkar SR, Stanek K, Colibaseanu DT (2022). Temporary vs. permanent stoma: factors associated with the development of complications and costs for rectal cancer patients. Int J Colorectal Dis.

[10] Joseph AL, Monkman H, Kushniruk A, Quintana Y (2023). Exploring patient journey mapping and the learning health system: scoping review. JMIR Hum Factors.

[11] Shu XP, Lv Q, Li ZW, Liu F, Liu XR, Li LS (2024). Does one-stitch method of temporary ileostomy affect the stoma-related complications after laparoscopic low anterior resection in rectal cancer patients?. Eur J Med Res.

[12] Ren Z, Tong L, Jin S, Wang Y, Xiao Q (2024). Nurses’ experiences of internet + nursing service based on service quality: a qualitative study. Stud Health Technol Inform.

[13] Li JH, Zhu YM, Zhu LH, Li XY (2024). Research progress on hospital excellent service. Chin Nurs Manag.

[14] Bulto LN, Davies E, Kelly J, Hendriks JM (2024). Patient journey mapping: emerging methods for understanding and improving patient experiences of health systems and services. Eur J Cardiovasc Nurs.

